# Supplementary material for: Nano-scale collinear multi-Q states driven by higher-order interactions
Source: Nat Commun. 2022 Sep 30;13:5764. doi: 10.1038/s41467-022-33383-w (PMC9525597; doi:10.1038/s41467-022-33383-w)
Supplement: Supplementary file 1 — Supplementary Information [file 41467_2022_33383_MOESM1_ESM.pdf]

Supplementary Information for: Nano-scale collinear multi-Q states driven by higher-order interactions

Mara Gutzeit,[1] André Kubetzka,[2] Soumyajyoti Haldar,[1] Henning Pralow,[1] Moritz A. Goerzen,[1] Roland Wiesendanger,[2] Stefan Heinze,[1] and Kirsten von Bergmann[2]

[1]*Institute of Theoretical Physics and Astrophysics, University of Kiel, Leibnizstrasse 15, 24098 Kiel, Germany*

[2]*Department of Physics, University of Hamburg, 20355 Hamburg, Germany*

Supplementary Table 1 | Calculated interlayer distances for Fe/n-Rh/Ir(111) systems.

Supplementary Table 2 | Heisenberg exchange constants calculated via DFT for Fe/n-Rh/Ir(111) systems.

Supplementary Table 3 | Higher-order exchange constants for Fe monolayers on different surfaces.

Supplementary Table 4 | Dzyaloshinskii-Moriya interaction constants and MAE calculated via DFT for Fe/n-Rh/Ir(111) systems.

Supplementary Table 5 | DFT energies for different magnetic states for varying Ir substrate layers of Fe/Rh/Rh/Ir(111).

Supplementary Table 6 | Details on the DFT calculations of higher-order exchange constants for Fe/n-Rh/Ir(111) systems.

Supplementary Figure 1 | SP-STM simulations for hexagonal spin states in hcp-Fe/n-Rh/Ir(111).

Supplementary Figure 2 | SP-STM simulations for uniaxial spin states in fcc-Fe/n-Rh/Ir(111).

Supplementary Figure 3 | STM and SP-STM images calculated via DFT for the uudd $\frac{1}{2}\overline{\Gamma\mathrm{M}}$ state of fcc-Fe/Rh/Rh/Ir(111).

Supplementary Figure 4 | Magnetic superstructures of Fe monolayers on hcp-Rh/Ir(111).

Supplementary Figure 5 | Magnetic superstructures of Fe monolayers on different Rh double-layers on Ir(111).

Supplementary Figure 6 | Magnetic superstructures of Fe monolayers on Rh1, Rh2 and Rh3.

Supplementary Figure 7 | Measurements with out-of-plane and in-plane sensitive tips.

Supplementary Figure 8 | DFT total energies for various spin structures in Fe/Rh/Rh/Ir(111).

Supplementary Figure 9 | Comparison of energy dispersions of Fe/n-Rh/Ir(111) for different numbers of Ir substrate layers.

Supplementary Figure 10 | Energy contribution to spin spiral energy dispersions due to SOC for Fe/n-Rh/Ir(111) films with different numbers of Ir substrate layers.

Supplementary Figure 11 | Comparison of layer-resolved energy contributions to spin spiral energy dispersions due to spin-orbit coupling (SOC) of Fe/n-Rh/Ir(111) systems for the case of 9 Ir substrate layers.

Supplementary Figure 12 | Comparison between atomistic spin model and DFT for the transformation of the uudd $\frac{1}{2}\overline{\Gamma\mathrm{M}}$ state into the 90° spin spiral calculated for fcc-Fe/Rh/Ir(111).

Supplementary Figure 13 | Spin model vs. DFT energies for selected magnetic states in Fe/Rh/Rh/Ir(111).

Supplementary Figure 14 | Energy contributions to SkX and MS states for hcp-Fe/Rh/Ir(111).

Supplementary Figure 15 | Energy contributions to uniaxial collinear vs. non-collinear spin states for hcp-Fe/Rh/Rh/Ir(111).

Supplementary Figure 16 | Energy of canted 12:15-MS states obtained via the atomistic spin model for hcp-Fe/Rh/Ir(111).

Supplementary Figure 17 | Energy of canted 4.67-atom states obtained via the atomistic spin model for fcc-Fe/Rh/Ir(111).

System	d_{FeRh} [Å]	d_{RhIr} [Å]	d_{IrIr} [Å]
fcc-Fe/Rh/Ir(111)	2.087	2.276	2.252
hcp-Fe/Rh/Ir(111)	2.079	2.258	2.258
System	d_{FeRh} [Å]	d_{RhRh} [Å]	d_{RhIr} [Å]
fcc-Fe/Rh/Rh/Ir(111)	2.076	2.272	2.224
hcp-Fe/Rh/Rh/Ir(111)	2.046	2.230	2.220

Supplementary Table 1 | Calculated interlayer distances for Fe/n-Rh/Ir(111) systems. Relaxed structural parameters for Fe/Rh/Ir(111) and Fe/Rh/Rh/Ir(111) in the ferromagnetic state and for both stackings of the Fe layer. Structural relaxations have been carried out with the `FLEUR` code by means of a symmetric slab with 9 Ir substrate layers and one Fe/Rh bilayer (Fe/Rh/Rh trilayer) on each side of the film.

System	J_1	J_2	J_3	J_4	J_5	J_6	J_7	J_8	J_9	J_{10}	J_{11}
fcc-Fe/Rh/Ir(111)	6.37	−1.50	−1.81	0.17	−0.02	0.11	−0.30	0.20	0.09	−0.28	0.03
	8.40	−1.96	−1.83	0.54	0.14	−0.08	−0.29	0.14	–	–	–
hcp-Fe/Rh/Ir(111)	5.14	−0.78	−1.84	0.39	0.00	−0.05	−0.17	−0.03	0.16	−0.06	0.03
	7.10	−1.37	−1.68	0.51	0.27	0.13	−0.26	0.15	–	–	–
fcc-Fe/Rh/Rh/Ir(111)	4.87	−1.65	−2.20	0.03	−0.02	0.07	−0.26	0.09	0.00	−0.22	−0.01
	7.54	−1.99	−2.16	0.34	0.04	−0.04	−0.26	0.18	−0.05	−0.05	0.04
hcp-Fe/Rh/Rh/Ir(111)	3.06	−0.93	−2.04	0.19	−0.03	−0.02	−0.16	−0.05	0.09	−0.05	0.01
	5.37	−1.27	−1.98	0.31	0.10	0.02	−0.17	0.08	−0.03	−0.04	0.02

Supplementary Table 2 | Heisenberg exchange constants calculated via DFT for Fe/n-Rh/Ir(111) systems. Heisenberg exchange constants (J_i) for Fe/Rh/Ir(111) and Fe/Rh/Rh/Ir(111) as extracted from fitting the respective spin spiral energy dispersion $E(\mathbf{q})$ neglecting HOIs. For every system the upper line lists the exchange constants obtained from calculations with 5 Ir layers in case of Fe/Rh/Ir(111) (cf. Fig. 4 of main paper and Supplementary Fig. 2) and 4 Ir layers in case of Fe/Rh/Rh/Ir(111) (cf. Extended Data Fig. 3 of the main paper and Supplementary Fig. 2) while the lower one shows the respective values obtained from a 9 Ir layer calculation (Supplementary Fig. 2) for comparison. All values are given in meV. Note, that the exchange constants J_1 to J_3 need to be modified according to Eqs. (5-7) given in the methods section of the main text if the HOI constants are included in the atomistic spin model given by Eq. (1).

System	B_1	Y_1	K_1
fcc-Fe/Rh/Ir(111)	2.86	4.31	1.30
hcp-Fe/Rh/Ir(111)	2.26	4.65	0.85
fcc-Fe/Rh/Rh/Ir(111)	2.84	4.45	1.07
hcp-Fe/Rh/Rh/Ir(111)	2.53	4.74	0.56
hcp-Fe/Rh(111) Ref. [1]	3.40	4.00	0.10
fcc-Fe/Ir(111) Refs. [1, 2]	−0.24	−0.24	−1.28

Supplementary Table 3 | Higher-order exchange constants for Fe monolayers on different surfaces. Four-site four spin (K_1), biquadratic (B_1) and three-site four spin interaction (Y_1) constants for Fe/n-Rh/Ir(111) systems. Values for hcp-Fe/Rh(111) are taken from Ref. [1] and for fcc-Fe/Ir(111) from Ref. [1, 2]. The exchange constants as well as the DMI and the MAE are listed in Supplementary Tables 2 and 3. All values are given in meV.

System	D_1	D_2	D_3	D_4	D_5	K_u
fcc-Fe/Rh/Ir(111)	0.42	−0.04	0.21	−0.03	0.09	−0.49
	0.49	−0.09	0.24	−0.05	0.13	
hcp-Fe/Rh/Ir(111)	0.71	0.01	0.03	0.04	0.04	−0.18
	0.79	0.07	0.04	0.03	0.01	
fcc-Fe/Rh/Rh/Ir(111)	0.58	−0.15	−0.01	−0.04	−0.01	−0.98
	0.51	−0.18	−0.06	−0.01	0.01	
hcp-Fe/Rh/Rh/Ir(111)	0.57	−0.10	−0.06	0.00	0.00	−0.87
	0.42	−0.05	−0.12	0.00	−0.04	

Supplementary Table 4 | Dzyaloshinskii-Moriya interaction constants and MAE calculated via DFT for Fe/n-Rh/Ir(111) systems. Dzyaloshinskii-Moriya interaction constants (D_i) and magnetocrystalline anisotropy energy constant (K_u) for Fe/Rh/Ir(111) and Fe/Rh/Rh/Ir(111). A negative value of K_u denotes an out-of-plane easy magnetization axis. For every system the upper line lists the values obtained from a calculation with 5 (4) Ir layers, the lower one the respective values from a 9 Ir layer calculation for comparison (see Supplementary Fig. 3). All values are given in meV.

System	number of Ir layers	uudd $\frac{1}{2}\overline{\Gamma\text{M}}$ (`FLEUR`)	uudd $\frac{1}{2}\overline{\Gamma\text{M}}$ (`VASP`)	12:15-MS	7:12-MS
fcc-Fe	4	−34.12	−31.53	−29.85	−29.60
	5	−23.06	−23.79	−21.09	−21.69
hcp-Fe	4	−32.75	−29.54	−29.82	−29.72
	5	−27.35	−26.85	−25.75	−25.48

Supplementary Table 5 | DFT energies for different magnetic states for varying Ir substrate layers of Fe/Rh/Rh/Ir(111). Comparison of DFT energies for the uudd $\frac{1}{2}\overline{\Gamma\text{M}}$ state (`FLEUR` and `VASP`), hexagonal 12:15-MS (`VASP`) and 7:12-MS state (`VASP`) for both stackings of the Fe layer on Fe/Rh/Rh/Ir(111) in case of 4 and 5 Ir substrate layers. While the energies for the uudd $\frac{1}{2}\overline{\Gamma\text{M}}$ states differ by up to 3 meV between `FLEUR` and `VASP` for an Ir substrate composed of only 4 layers, they reduce to 0.7 meV by increasing the layer thickness to 5 and can hence be regarded as identical similar to the case of Fe/Rh/Ir(111). Likewise the hexagonal 12:15- and 7:12-MS state remain almost energetically degenerate upon increasing the substrate thickness. All energies are given with respect to the ferromagnetic state in meV/Fe atom.

System	K_1	B_1	Y_1	$\Delta E^{uudd}_{\overline{\text{M}}/2}$	$\Delta E^{uudd}_{3\overline{\text{K}}/4}$	$\Delta E^{3Q}_{\overline{\text{M}}}$
fcc-Fe/Rh/Ir(111)	1.30	2.86	4.31	−18.28	16.20	6.16
	1.05	2.74	4.20	−19.38	14.24	3.38
hcp-Fe/Rh/Ir(111)	0.85	2.26	4.65	−20.78	16.35	−3.64
	0.75	2.72	4.48	−22.75	13.09	−1.36
fcc-Fe/Rh/Rh/Ir(111)	1.07	2.84	4.45	−20.54	15.02	2.87
hcp-Fe/Rh/Rh/Ir(111)	0.56	2.53	4.74	−24.57	13.34	−5.81

Supplementary Table 6 | Details on the DFT calculations of higher-order exchange constants for Fe/n-Rh/Ir(111) systems. Four-site four spin (K_1), biquadratic (B_1) and three-site four spin (Y_1) interaction constants for Fe/Rh/Ir(111) and Fe/Rh/Rh/Ir(111). ΔE denotes the energy difference between the multi-Q and the corresponding single-Q spin spiral state, respectively i.e., $\Delta E^{uudd}_{\overline{\text{M}}/2}= E^{uudd}_{\overline{\text{M}}/2} - E^{1\text{Q}}_{\overline{\text{M}}/2}$, $\Delta E^{uudd}_{3\overline{\text{K}}/4}= E^{uudd}_{3\overline{\text{K}}/4} - E^{1\text{Q}}_{3\overline{\text{K}}/4}$ and $\Delta E^{3\text{Q}}_{\overline{M}}= E^{3\text{Q}}_{\overline{\text{M}}} - E^{1\text{Q}}_{\overline{\text{M}}}$. The HOI terms are calculated according to Eqs. (2)-(4) in the main part of the paper. For both stackings of the Fe layer in Fe/Rh/Ir(111) the upper line lists the values obtained from a `FLEUR` calculation with 5 Ir layers, the lower one the respective values from a 9 Ir layer `FLEUR` calculation for comparison. All values are given in meV.

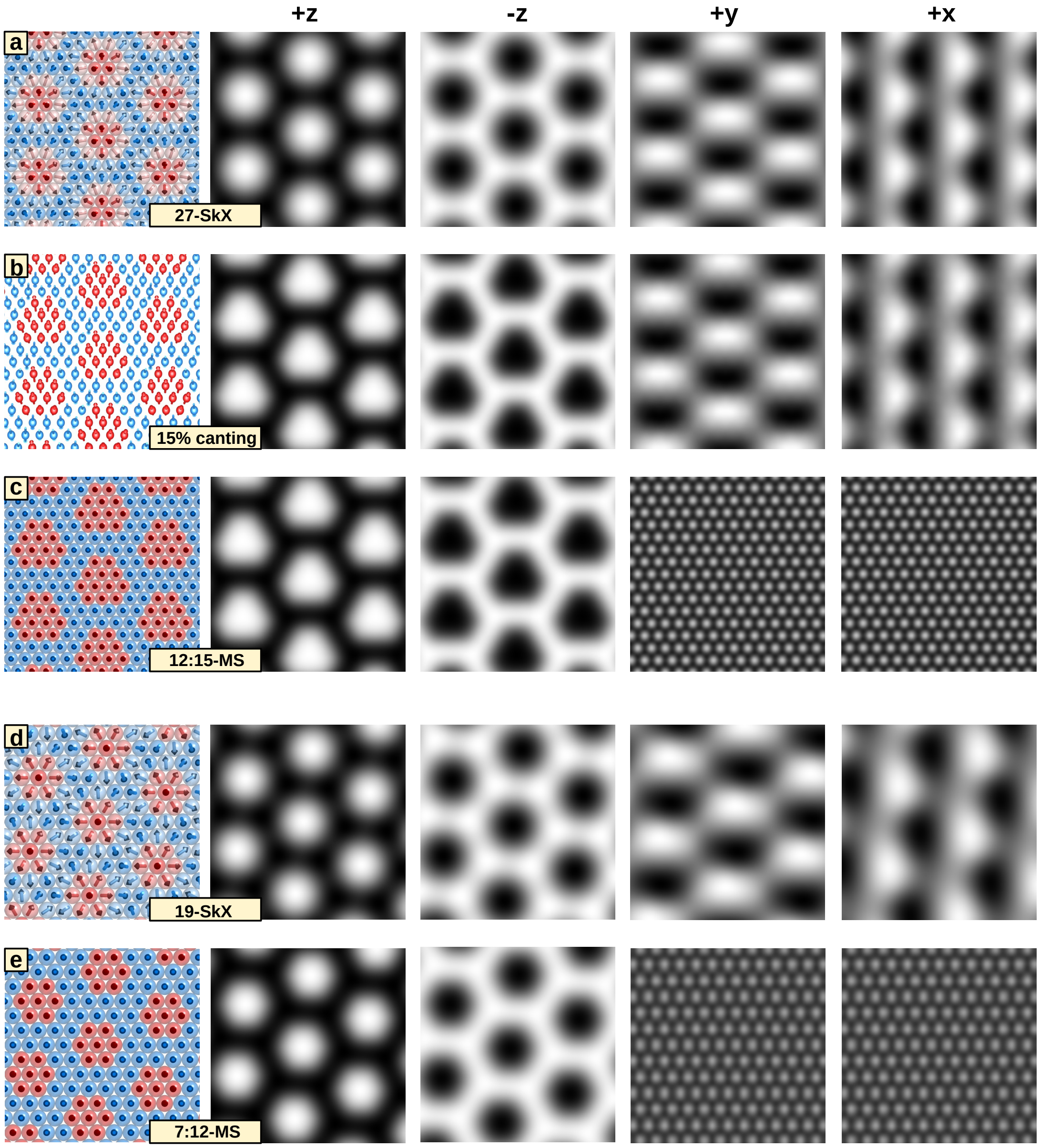

Supplementary Figure 1 |SP-STM simulations for hexagonal spin states in hcp-Fe/n-Rh/Ir(111). a-c Sketches of the 27-SkX state, the energetically lowest 15% canted 12:15-MS state, and the collinear 12:15-MS state of hcp-Fe/Rh/Ir(111) and **d-e** the 19-SkX and collinear 7:12-MS state of hcp-Fe/Rh/Rh/Ir(111) followed by the corresponding simulated SP-STM images of the same area with a tip magnetization in $+z$, $-z$ (out-of-plane) as well as $+y$ and $+x$ direction (in-plane) with 50% spin polarization in each case. The SP-STM simulations have been performed based on the model described in Ref. [3] at a height of 6 Å. The corrugation amplitudes for the 27-SkX state in **a** amount to 37 pm for a tip magnetization along the z direction and to 29 pm for a tip magnetization along the x and y direction. For the 15% canted 12:15-MS state in **b** (and the collinear 12:15-MS state in **c**) the corrugation amplitude amounts to 43 pm for a tip magnetization along the z direction. Note, that although the SP-STM images for the 27-SkX and the 15% canted 12:15-MS state with in-plane magnetized tips ($+y$ and $+x$) are qualitatively the same, the corrugation amplitude for the 15% canted 12:15-MS state is only 5 pm and hence by a factor of 5.4 smaller than for the 27-SkX. The corrugation amplitude for a tip magnetization along the z direction of the 7:12-MS state **e** amounts to 44 pm, while the respective value for the non-collinear 19-SkX state in **d** is 32 pm (around 25 pm for a tip magnetization along x and y direction). The corrugation amplitudes for the images with in-plane magnetized tips of the collinear 12:15-MS state and the 7:12-MS state, which show the atomic lattice, amount to only 0.1 pm. This small value is a well-known quantitative deficiency of the Tersoff-Hamann model [4] for close-packed metal surfaces (see e.g. discussion in Ref. [5]).

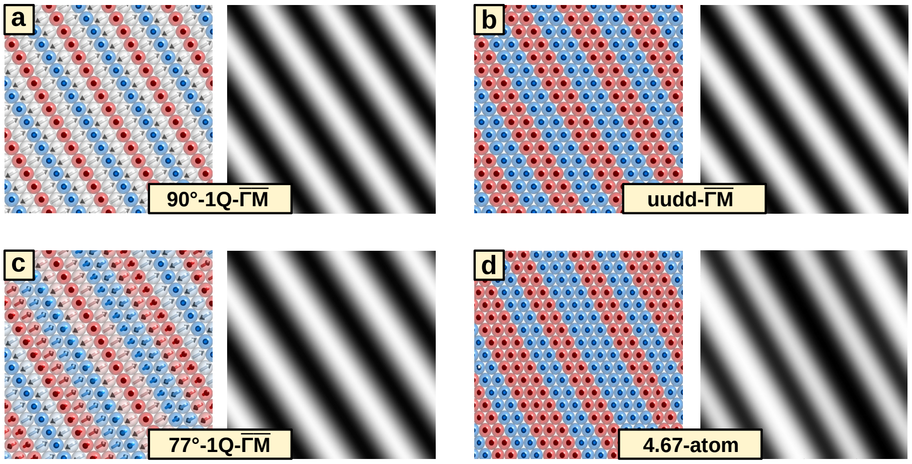

Supplementary Figure 2 |SP-STM simulations for uniaxial spin states in fcc-Fe/n-Rh/Ir(111) . a-d Sketches of the 90° spin spiral along $\overline{\Gamma M}$ direction, the uudd $\overline{\Gamma M}$ state, the 77° spin spiral along $\overline{\Gamma M}$ and the corresponding collinear 4.67-atom state followed by the respective simulated SP-STM images with a tip magnetization in $+z$ direction and 50% spin polarization in each case. The SP-STM simulations have been performed based on the model described in Ref. [3] at a height of 6 Å.

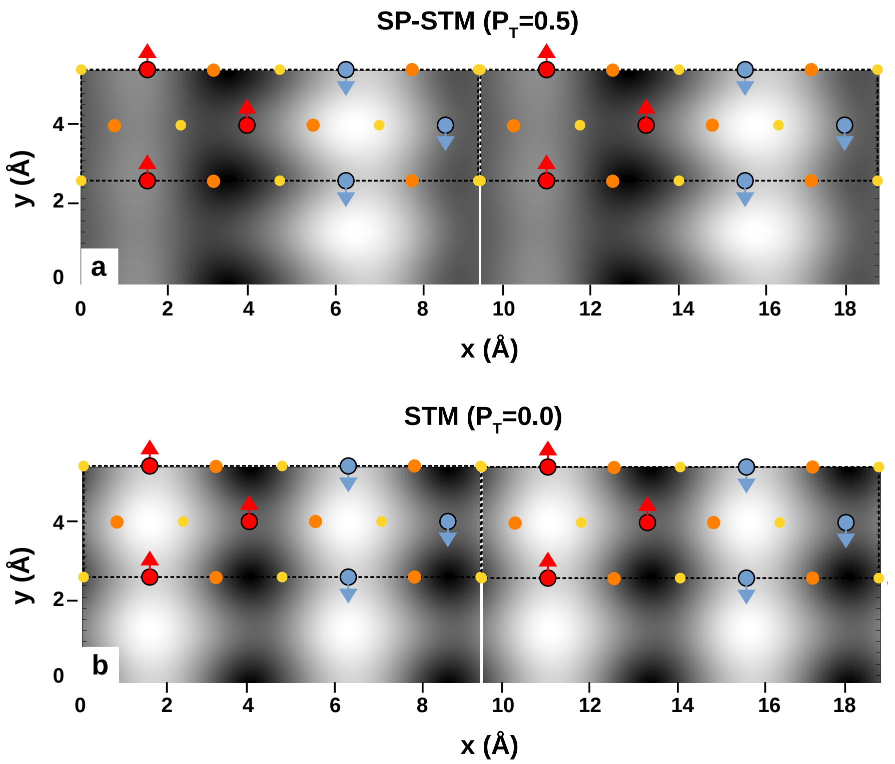

Supplementary Figure 3 | STM and SP-STM images calculated via DFT for the uudd $\frac{1}{2}\overline{\Gamma M}$ state of fcc-Fe/Rh/Rh/Ir(111) . **a** SP-STM image at 3 Å above the surface for a tip spin polarization of 0.5 and **b** STM image for a non-spin-polarized tip in the energy range [E_F,E_F+0.05 eV]. The calculations have been performed using the `FLEUR` code based on the spin-polarized generalization [6] of the Tersoff-Hamann model [4]. The red (blue) circles with upward (downward) pointing arrows denote Fe atoms with the respective magnetization direction while yellow (orange) circles depict atoms of the first (second) Rh layer as counted from the film surface. For easier visualization, the magnetization components of the Fe atoms are drawn in-plane; in the corresponding DFT calculation they are pointing along the out-of-plane easy magnetization direction instead.

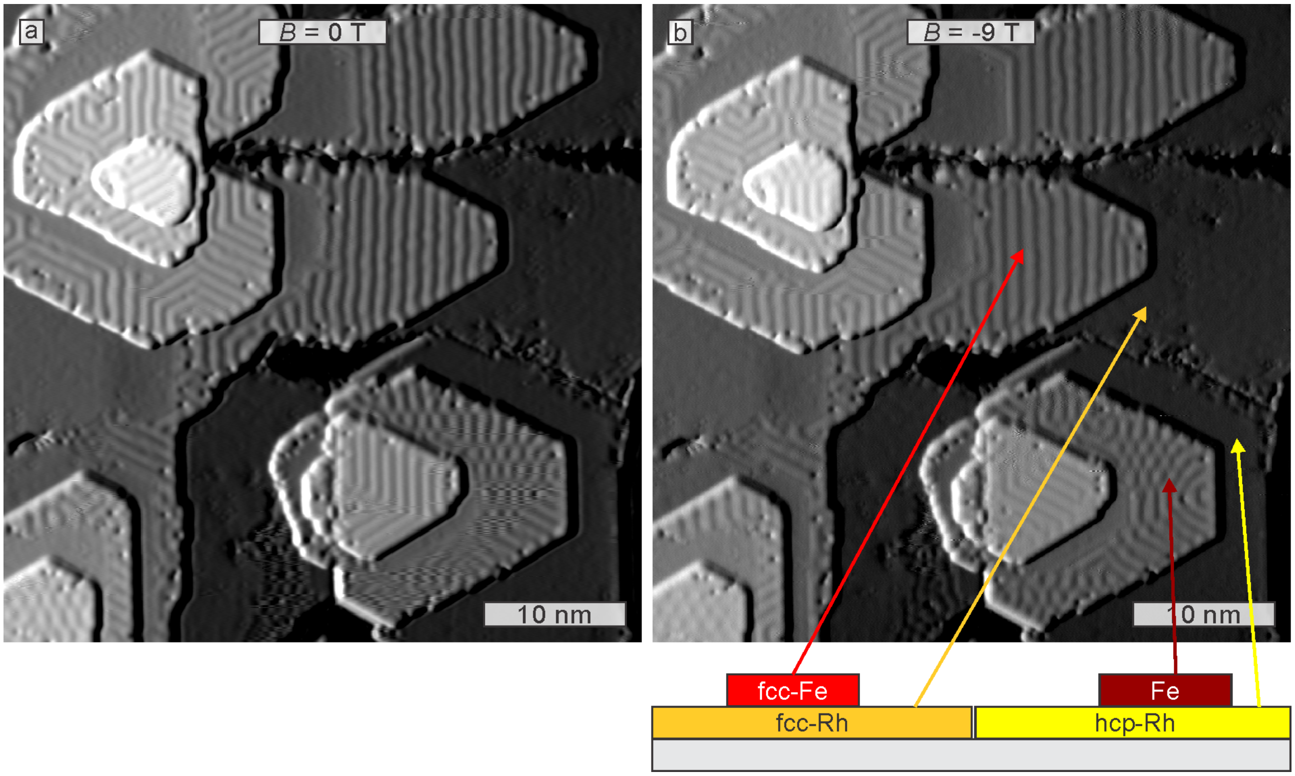

Supplementary Figure 4 | Magnetic superstructures of Fe monolayers on hcp-Rh/Ir(111). a,b, Partially differentiated constant-current measurements of a sample with submonolayer coverage of Rh and Fe on Ir(111) in zero magnetic field and at -9 T, respectively. The Fe-ML indicated in the bottom right has grown on one of the rare hcp-Rh/Ir(111) islands. The magnetic signal reveals the coexistance of uniaxial and two-dimensionally modulated spin textures within this specific Fe monolayer; the applied magnetic field of -9 T appears to stabilize the local magnetic texture. (Measurement parameters: $U = +30$ mV, $I = 2.5$ nA, B as indicated, $T = 4.2$ K, Cr-bulk tip).

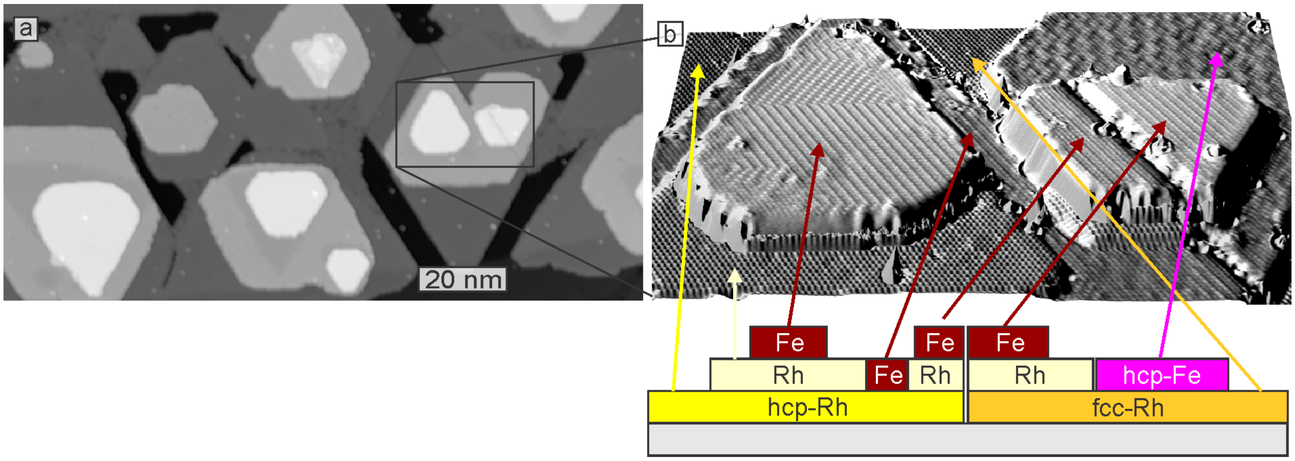

Supplementary Figure 5 | Magnetic superstructures of Fe monolayers on different Rh double-layers on Ir(111). a, Overview constant-current measurement of a sample with submonolayer coverage of Rh and Fe on Ir(111). **b,** Perspective view of topographic and current signal of the area indicated in a. Also the Fe-ML on a Rh double-layer with hcp-Rh in the bottom layer exhibits an *uudd*-state. (Measurement parameters: a, $U = +15$ mV, $I = 3.3$ nA; b, $U = +34$ mV, $I = 5.3$ nA; both: $B = -4$ T, $T = 4.2$ K, Cr-bulk tip).

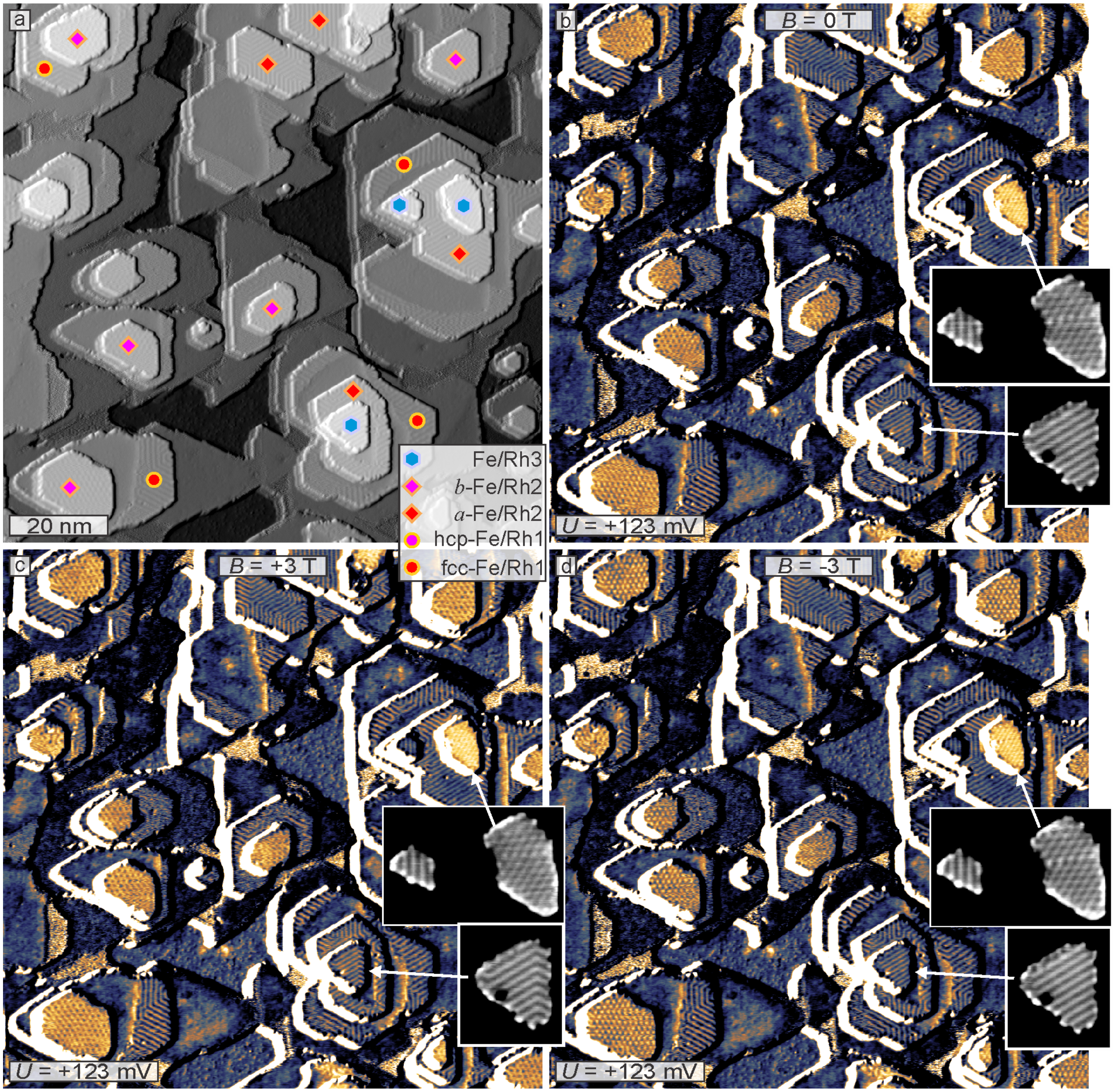

Supplementary Figure 6 | Magnetic superstructures of Fe monolayers on Rh1, Rh2 and Rh3. a,b, Partially differentiated constant-current measurement and simultaneously obtained map of differential conductance dI/dU, respectively. The colored dots in the topographic image indicate the different layer sequences of Fe/Rh; the blue hexagons refer to Fe monolayers on three layers of Rh. Whereas the two smaller Fe/Rh3 islands with the darker dI/dU signal likely host an *uudd*-state, the larger Fe/Rh3 island with the brighter dI/dU signal exhibits a hexagonal state with a slightly reduced lattice constant compared to *b*-Fe/Rh2; see inset zoomed topographic image (raw data with $\Delta z = \pm 10\,\mathrm{pm}$) for better visibility of the symmetry of the magnetic state. **c,d,** dI/dU-maps at $B = +3\,\mathrm{T}$ and $B = -3\,\mathrm{T}$, respectively (all dI/dU maps have identical color scales). (Measurement parameters: $U = +123\,\mathrm{mV}$, $I = 0.94\,\mathrm{nA}$; B as indicated, $T = 4.2\,\mathrm{K}$, Cr-bulk tip).

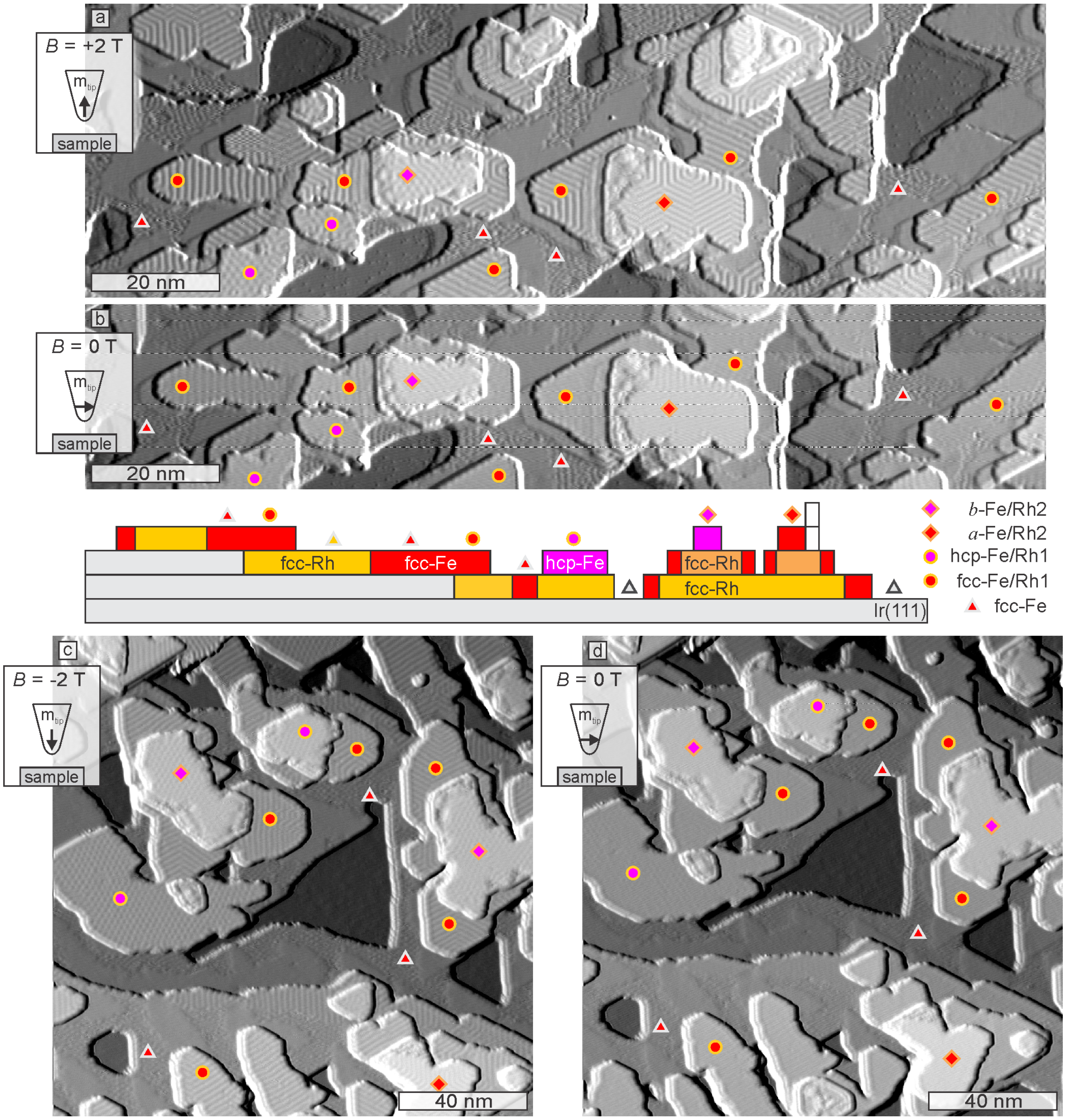

Supplementary Figure 7 | Measurements with out-of-plane and in-plane sensitive Fe-coated W tips. a,b, The same sample area measured with an Fe-coated W tip with and without applied out-of-plane magnetic field; Fe tips are typically sensitive to the in-plane sample magnetization at $B = 0\,\mathrm{T}$, whereas an applied magnetic field aligns the tip magnetization, resulting in a tip sensitive to the out-of-plane components, see sketches to the left. Whereas the magnetic corrugation stemming from the out-of-plane sample magnetization components is strong for all Fe monolayer areas in a, the magnetic contrast observed in b is hardly visible on the Fe monolayer in contact with Rh; however, the Fe monolayer directly on Ir(111) shows magnetic contrast in both cases, demonstrating that the tip is spin-polarized in both measurements, regardless of the tip change that occurred in between them. **c,d,** Same as a,b, but for a different sample area. These experiments suggest that on all Fe monolayers that are in contact with Rh, the out-of-plane magnetization components are much more prominent compared to the in-plane sample magnetization components. A quantitative evaluation is not possible, first because other magnetoresistance effects can play a role, and second because of the tip changes between images. (Measurement parameters: $U = +50\,\mathrm{mV}$, $I = 3\,\mathrm{nA}$; B as indicated, $T = 8.3\,\mathrm{K}$, Fe-coated W tip).

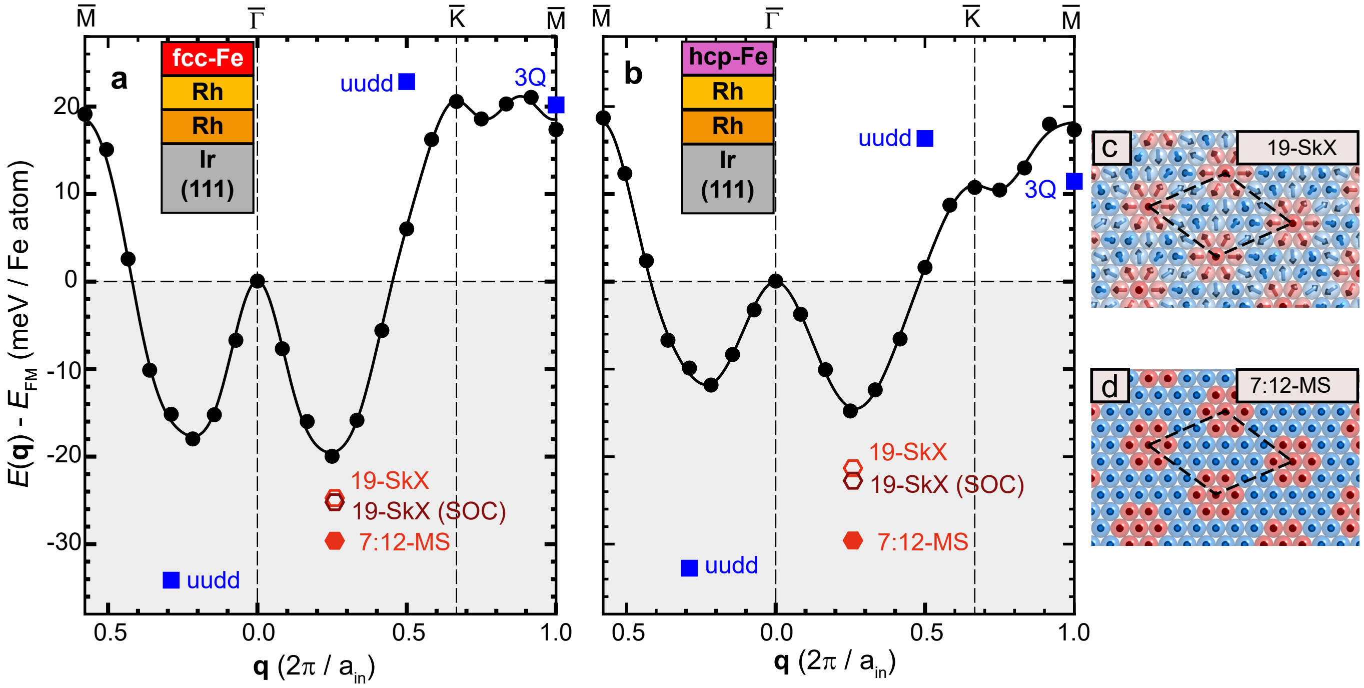

Supplementary Figure 8 DFT total energies for various spin structures in Fe/Rh/Rh/Ir(111). The energy dispersion $E(\mathbf{q})$ of flat cycloidal spin spirals is shown for **a** fcc-Fe/Rh/Rh/Ir(111) and **b** hcp-Fe/Rh/Rh/Ir(111) along the two high symmetry directions of the two-dimensional Brillouin zone. The black circles denote DFT total energies including spin-orbit coupling (SOC), i.e. the DMI and MAE. Black lines represent a fit to the Heisenberg model including the contributions of DMI and MAE. The energies of the two *uudd* states and the 3Q state are marked by blue squares at the **q**-values of the respective 1Q states. The skyrmion lattice (SkX) states are marked by open symbols. Sketches in panels **c,d** show the spin structures including the unit cells. For *uudd* and 3Q spin structures see Fig.4d-f in the main text. Here, we find the same preference of collinear magnetic order for the Fe/Rh2 system (as observed in Fe/Rh1 system), where the considered collinear 7:12-MS state has a significantly lower energy than the non-collinear 19-SkX counterpart. The *uudd* state seems to be the ground state for both stacking, however, in hcp-Fe/Rh2, for which the 19 atom unit cell has been observed experimentally, the 7:12-MS is very close in energy (cf. values given in Supplementary Table 4).

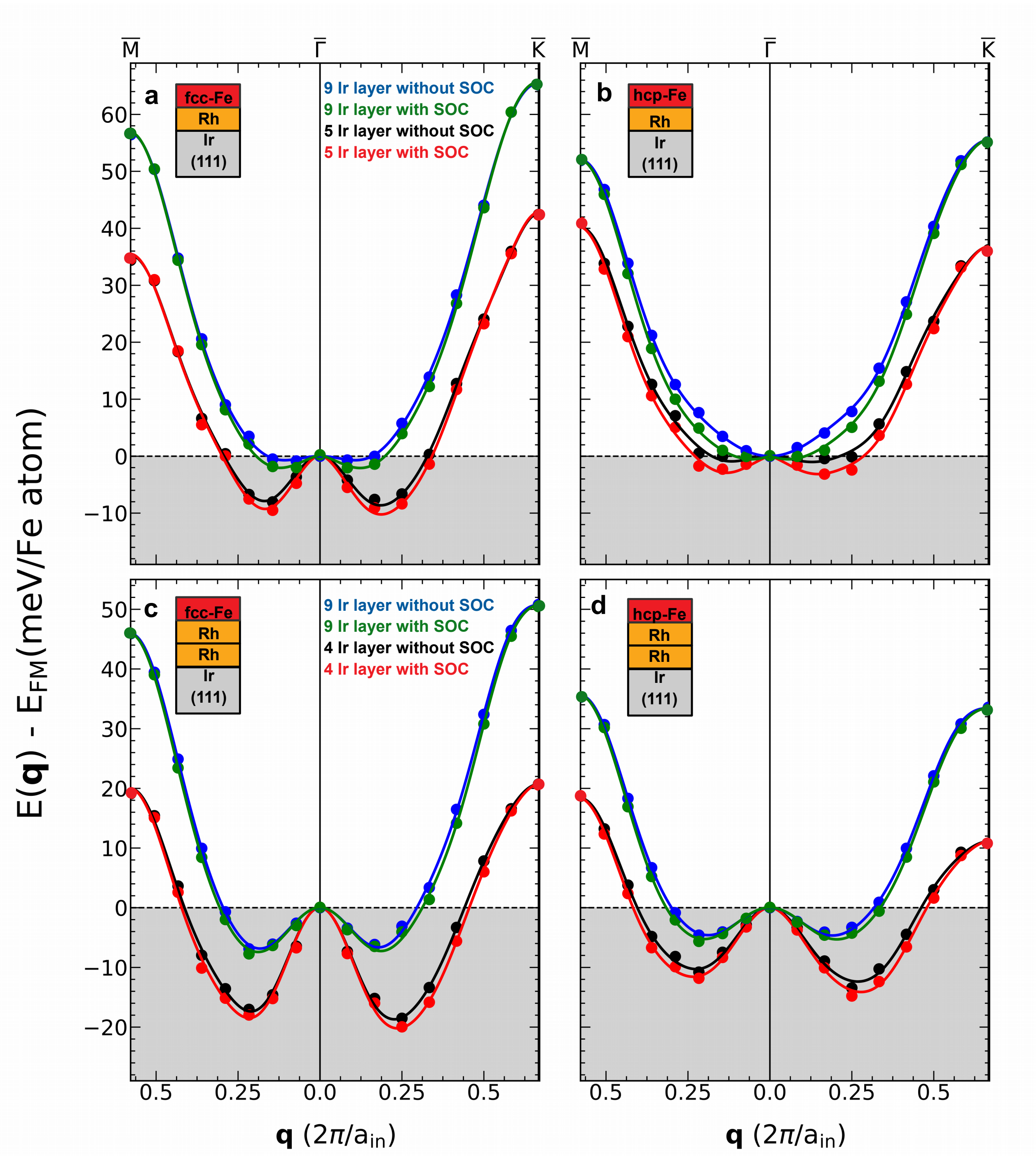

Supplementary Figure 9 | Comparison of energy dispersions of Fe/n-Rh/Ir(111) for different numbers of Ir substrate layers. **a** Energy dispersion E(**q**) of flat spin spirals for fcc-Fe/Rh/Ir(111), **b** hcp-Fe/Rh/Ir(111), **c** fcc-Fe/Rh/Rh/Ir(111) and **d** hcp-Fe/Rh/Rh/Ir(111) calculated by means of DFT along the two high symmetry directions of the two-dimensional Brillouin zone. Blue (black) circles denote scalar-relativistic DFT energies, whereas the blue (black) lines represent a fit to the Heisenberg model for a substrate thickness of 9 (5 for Fe/Rh/Ir(111) and 4 for Fe/Rh/Rh/Ir(111)) Ir layers. Green (red) circles indicate DFT energies including the effect of spin-orbit coupling (SOC), i.e. the DMI and MAE, while green (red) lines represent the fit to the Heisenberg model shifted by the effects of SOC for a substrate thickness of 9 (5 for Fe/Rh/Ir(111) and 4 for Fe/Rh/Rh/Ir(111)) Ir layers .

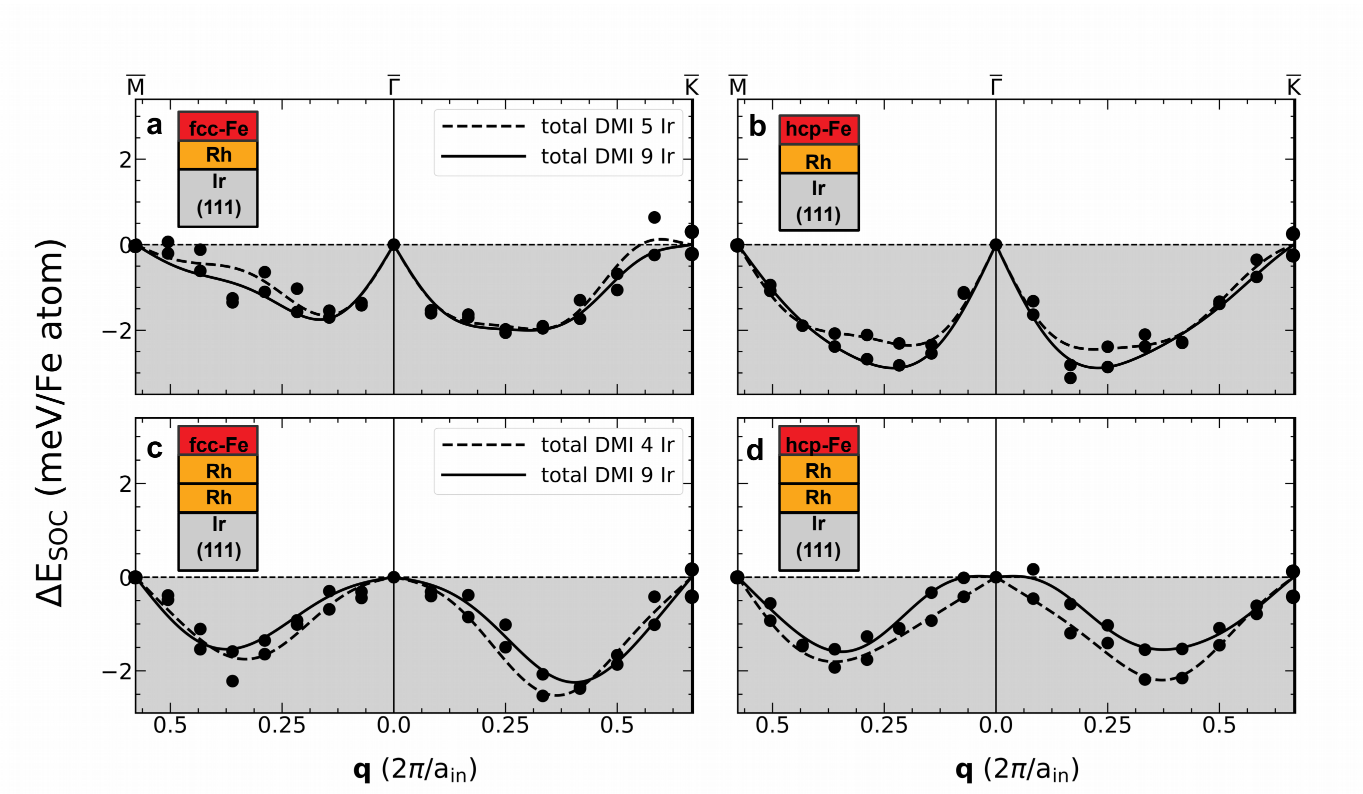

Supplementary Figure 10 | Energy contribution to spin spiral energy dispersions due to SOC for Fe/n-Rh/Ir(111) films with different numbers of Ir substrate layers. **a** Energy contribution due to spin-orbit coupling (SOC), $\Delta E_{\mathrm{SOC}}(\mathbf{q})$, to the energy dispersion of spin spirals for fcc-Fe/Rh/Ir(111), **b** hcp-Fe/Rh/Ir(111), **c** fcc-Fe/Rh/Rh/Ir(111) and **d** hcp-Fe/Rh/Rh/Ir(111) calculated by means of DFT along the two high symmetry directions of the two-dimensional Brillouin zone. Black circles denote DFT energies, whereas the black filled (dashed) lines represent a fit to the Dzyaloshinskii-Moriya interaction (DMI) for a substrate thickness of 9 (5 for Fe/Rh/Ir(111) and 4 for Fe/Rh/Rh/Ir(111)) Ir layers.

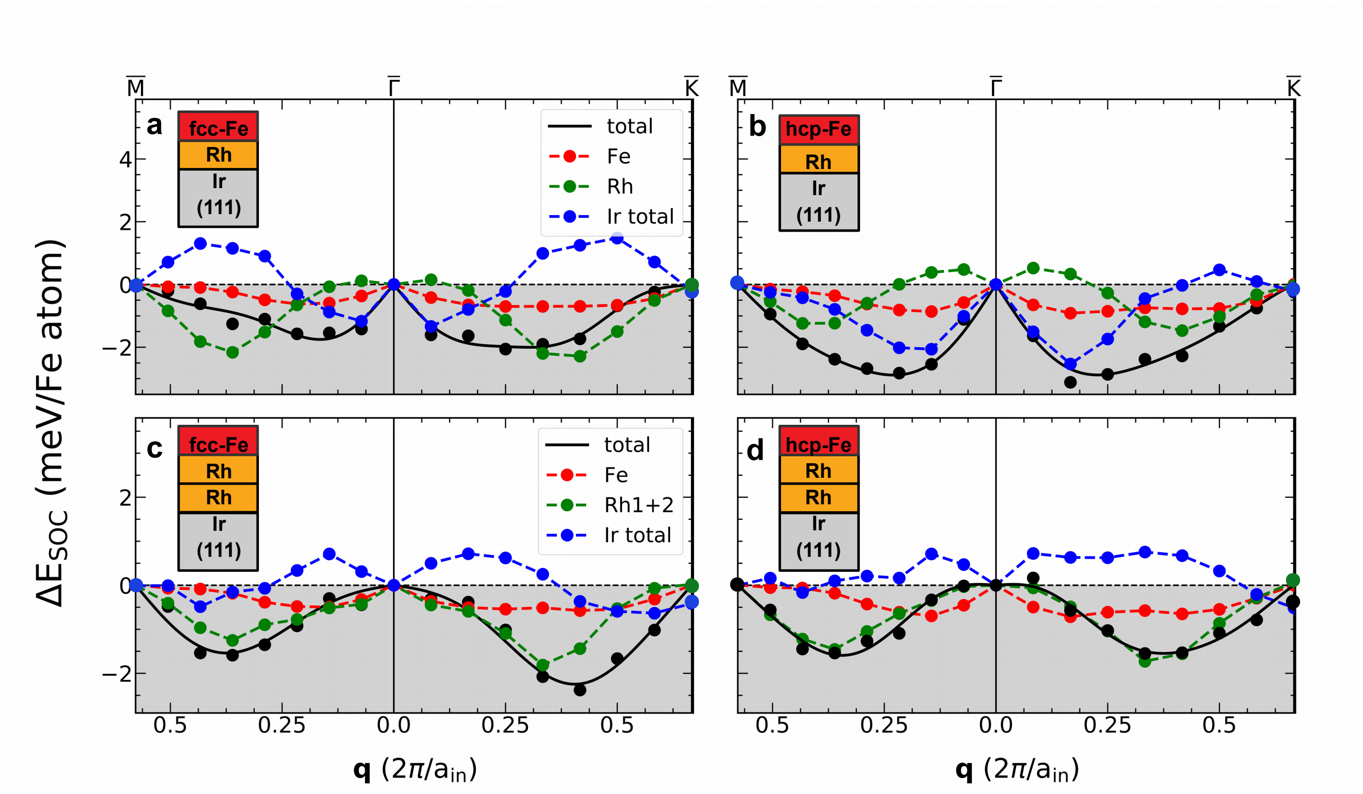

Supplementary Figure 11 | Comparison of layer-resolved energy contributions to spin spiral energy dispersions due to spin-orbit coupling (SOC) of Fe/n-Rh/Ir(111) systems for the case of 9 Ir substrate layers. **a** Layer-resolved SOC energy contributions, $\Delta E_{\mathrm{SOC}}(\mathbf{q})$, for fcc-Fe/Rh/Ir(111), **b** hcp-Fe/Rh/Ir(111), **c** fcc-Fe/Rh/Rh/Ir(111) and **d** hcp-Fe/Rh/Rh/Ir(111) calculated by means of DFT along the two high symmetry directions of the two-dimensional Brillouin zone. Black circles denote total DFT energies, whereas the black filled lines represent a fit to the Dzyaloshinskii-Moriya interaction (DMI). The dashed lines connecting the data points for Fe (blue), Rh (green) and the sum of the Ir layers (blue) serve as a guide to the eye.

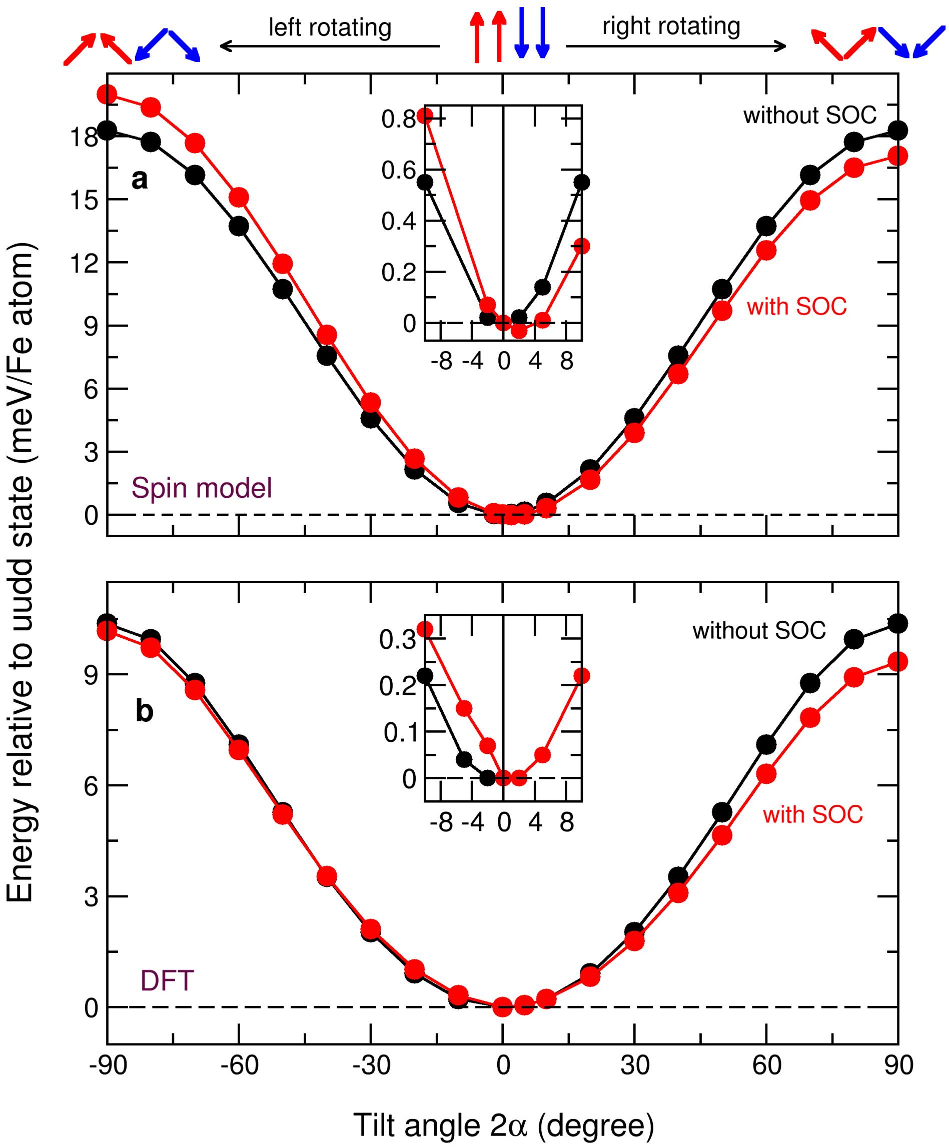

Supplementary Figure 12 | Comparison between atomistic spin model and DFT for the transformation of the uudd $\frac{1}{2}\overline{\Gamma\mathrm{M}}$ state into the 90° spin spiral calculated for fcc-Fe/Rh/Ir(111). Continuous transformation of the uudd state into the 90° clockwise (anticlockwise) spin spiral with and without spin-orbit coupling effects calculated via **a** the atomistic spin model and **b** via DFT for fcc-Fe/Rh/Ir(111) (5 Ir substrate layers). The canting angle α measures the deviation from the easy out-of-plane magnetization axis; hence, the uudd state corresponds to α=0° and the 90° spin spiral to α=45°. Negative values of the tilt angle α refer to an anticlockwise rotation of the magnetic moments. The lines connecting the data points serve as a guide to the eye.

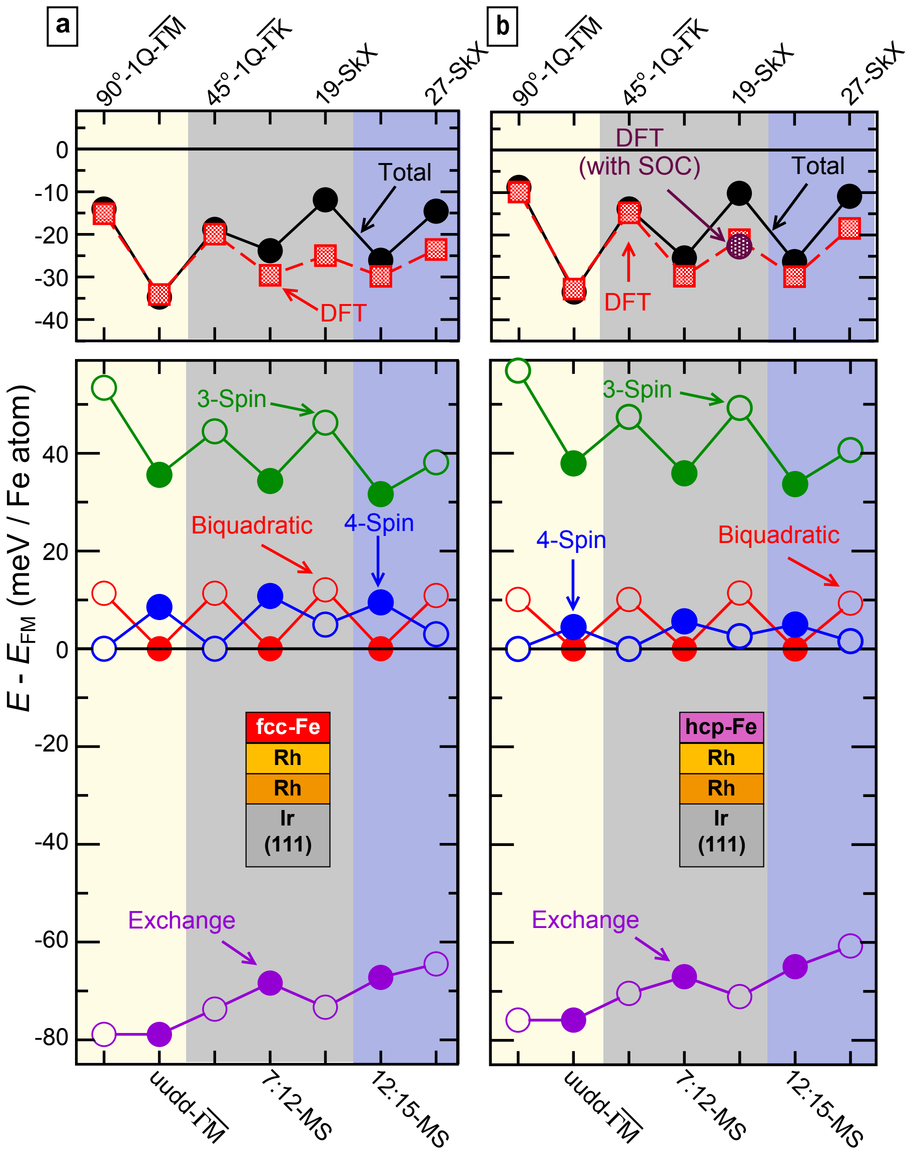

Supplementary Figure 13 | Spin model vs. DFT energies for selected magnetic states in Fe/Rh/Rh/Ir(111). **a**,**b**, Upper panels show the total energies for fcc-Fe/Rh/Rh/Ir(111) and hcp-Fe/Rh/Rh/Ir(111), respectively, with respect to the FM reference state; red squares are DFT values (violet dots include spin-orbit coupling), black circles are obtained via the atomistic spin model with DFT parameters for the magnetic interactions. On the upper axis the non-collinear states and on the lower axis the corresponding collinear states are specified (background colors serve to group the states which have the same Q-vectors). In the lower panels the total energy of the spin model (black circles in upper panel) is decomposed into the contributions from the Heisenberg exchange, the two-site four spin interaction (biquadratic), the three-site four spin interaction (3-Spin) and the four-site four spin interaction (4-Spin). Filled (open) circles indicate collinear (non-collinear) states. Note that for the calculation of total energies within the spin model the DMI and the MAE were taken into account (not shown here). The lines connecting the data points serve as a guide to the eye.

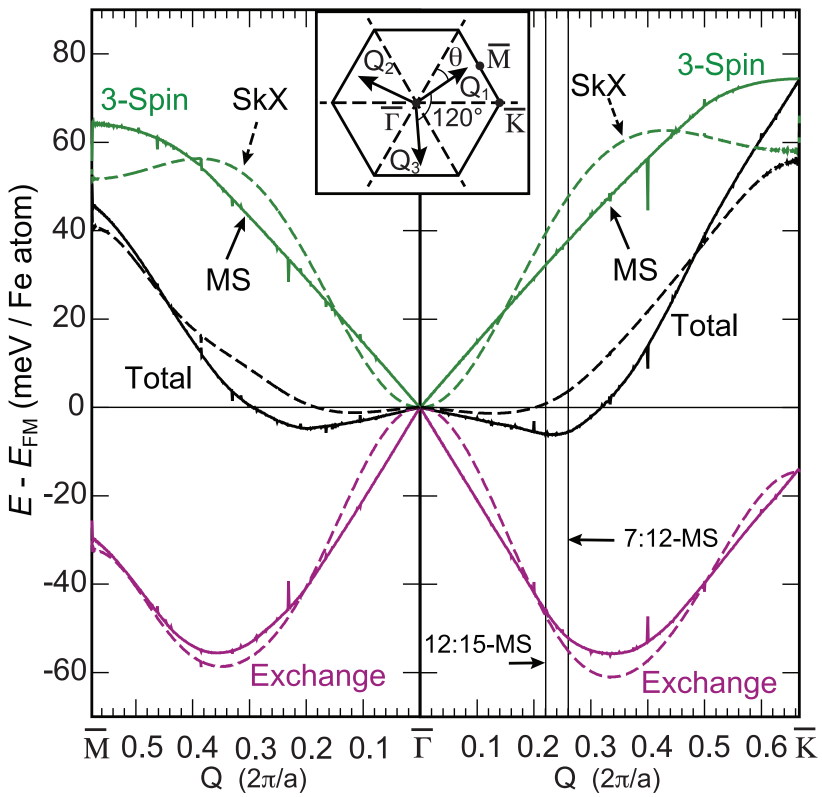

Supplementary Figure 14 | Energy contributions to SkX and MS states for hcp-Fe/Rh/Ir(111). Plot of the energy contributions from the exchange and three-site four spin interaction to the total energy $E(Q)$ for mosaic (MS) and skyrmion lattice (SkX) states for $\mathbf{Q}$ along the $\overline{\text{M}\Gamma\text{K}}$ direction. Energies were obtained in the spin model with DFT parameters of hcp-Fe/Rh/Ir(111). Note, that the energy contributions from the biquadratic, the four-site four spin interaction, the DMI, and the MAE are not displayed but included in the total energy. The spikes in the energy curves for the MS states originate from changes of the local spin structure on the discrete atomic lattice due to taking only the z-component of the magnetic moments in its construction (see methods). **Inset** Sketch of the 2D Brillouin zone with the three $\mathbf{Q}$-vectors: $\mathbf{Q_1}$, $\mathbf{Q_2}$ and $\mathbf{Q_3}$ used to obtain the SkX along the $\overline{\Gamma\text{M}}$ direction; to obtain the SkX for the $\overline{\Gamma\text{K}}$ direction the Q-vectors are rotated by an angle $\theta = 30°$ (see methods for details).

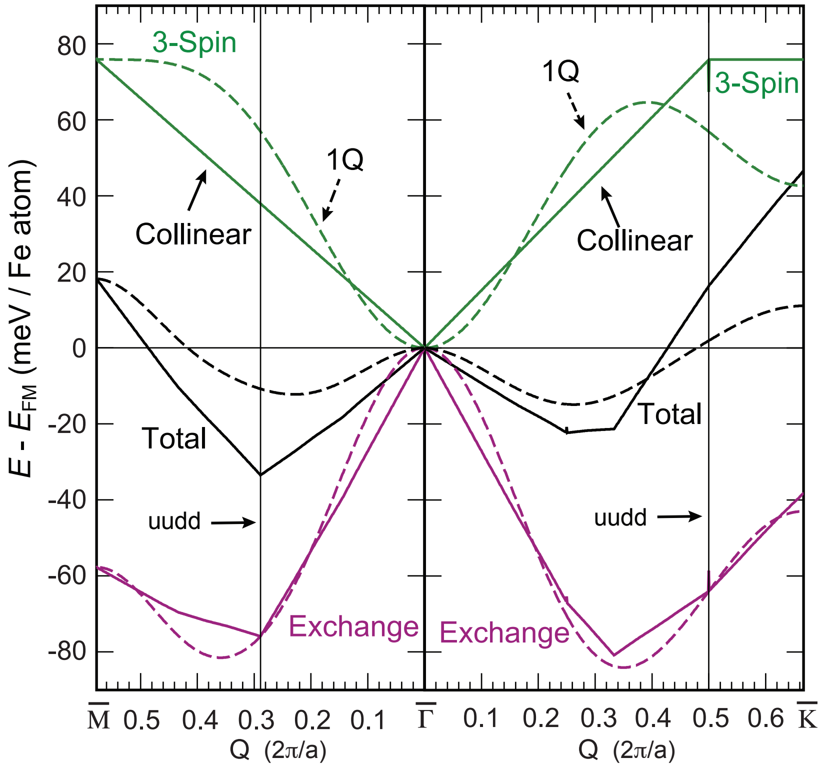

Supplementary Figure 15 | Energy contributions to uniaxial collinear vs. non-collinear spin states for hcp-Fe/Rh/Rh/Ir(111). Plot of the energy contributions from the exchange and three-site four spin interaction to the total energy $E(Q)$ along the $\overline{\mathrm{M}\Gamma\mathrm{K}}$ direction of the 2D BZ for uniaxial collinear states and the corresponding spin spiral (1Q) states from which they were constructed. The value of Q for the *uudd* states along both high symmetry directions are marked by lines. Energies were obtained in the spin model with DFT parameters of hcp-Fe/Rh/Rh/Ir(111). Note, that the energy from the biquadratic and the four-site four spin interaction is not displayed but included in the total energy.

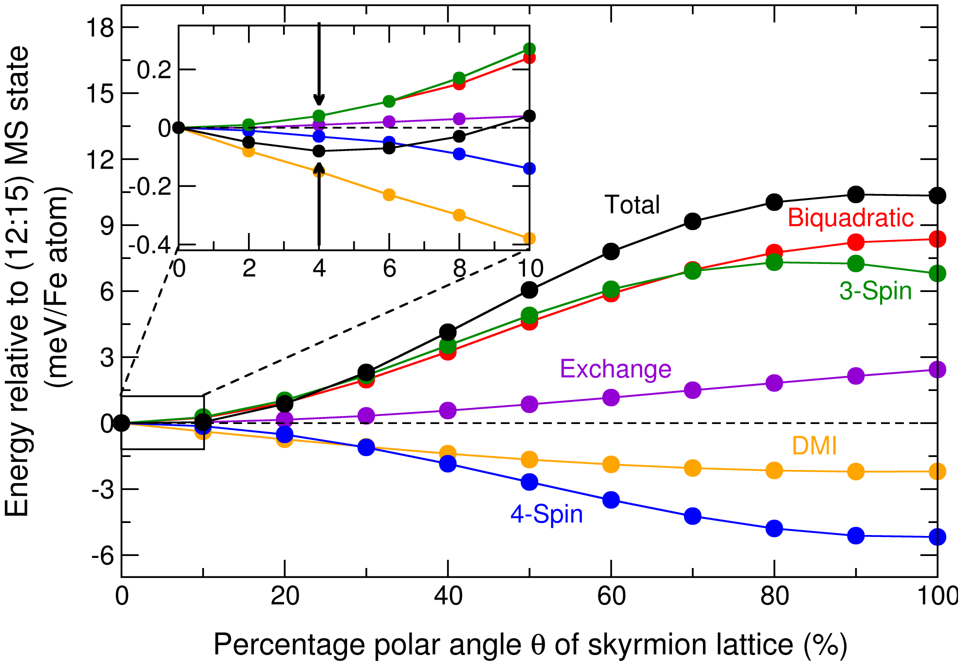

Supplementary Figure 16 | Energy of canted 12:15-MS states obtained via the atomistic spin model for hcp-Fe/Rh/Ir(111). Energy of the canted 12:15-MS state as a function of the proportional polar angle θ of the originally proposed hexagonal nanoskyrmion lattice resolved by the contributions from different magnetic interactions covered by the extended Heisenberg model. The proportional polar angle θ of every atom is defined as $\theta(x)=\theta_0 + x(\theta_f - \theta_0)$ with $x \in [0, 1]$ where the value x=0 is set for the collinear 12:15-MS state and x=1 for the fully noncollinear hexagonal skyrmion lattice. θ_f refers to the final value of every magnetic moment on the atoms in the nanoskyrmion lattice, whereas θ_0 is set to $0°$ for upward pointing moments ($180°$ for downward pointing moments). The in-plane angles ϕ of the atoms in the originally proposed skyrmion lattice are not changed with the variation of θ, but kept fixed to the values of the nanoskyrmion. Filled circles are obtained by means of the atomistic spin model with DFT interaction parameters from 5 layers of the Ir substrate, while lines serve as a guide to the eye.

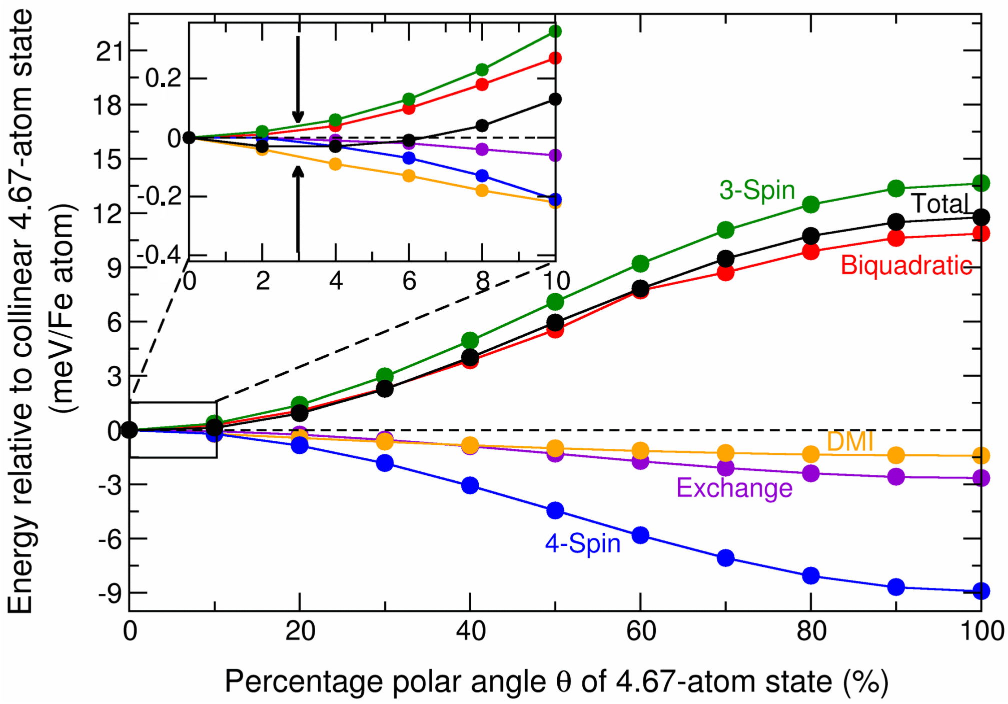

Supplementary Figure 17 | Energy of canted 4.67-atom states obtained via the atomistic spin model for fcc-Fe/Rh/Ir(111). Energy of the canted 4.67-atom state as a function of the proportional polar angle θ of the originally proposed 4.67-atomic 77.14° spin spiral state resolved by the contributions from different magnetic interactions covered by the extended Heisenberg model. The proportional polar angle θ of every atom is defined as $\theta(x)=\theta_0 + x(\theta_f - \theta_0)$ with $x \in [0, 1]$ where the value x=0 is set for the collinear state and x=1 for the fully noncollinear spin spiral. θ_f refers to the final value of every magnetic moment on the atoms of the spin spiral, whereas θ_0 is set to 0° for moments with upward pointing magnetization direction (180° for downward pointing magnetization direction). Filled circles are obtained by means of the atomistic spin model with DFT interaction parameters from 5 layers of the Ir substrate, while lines serve as a guide to the eye.

Supplementary References

[1] Krönlein, A. *et al.* Magnetic ground state stabilized by three-site interactions: Fe/Rh(111). *Phys. Rev. Lett.* **120**, 207202 (2018).
[2] Heinze, S. *et al.* Spontaneous atomic-scale magnetic skyrmion lattice in two dimensions. *Nat. Phys.* **7**, 713–718 (2011).
[3] Heinze, S. Simulation of spin-polarized scanning tunneling microscopy images of nanoscale non-collinear magnetic structures. *Appl. Phys. A* **85**, 407–414 (2006).
[4] Tersoff, J. & Hamann, D. R. Theory of the scanning tunneling microscope. *Phys. Rev. B* **31**, 805–813 (1985).
[5] Heinze, S., Blügel, S., Pascal, R., Bode, M. & Wiesendanger, R. Prediction of bias-voltage-dependent corrugation reversal for STM images of bcc (110) surfaces: W(110), Ta(110), and Fe(110). *Phys. Rev. B* **58**, 16432–16445 (1998).
[6] Wortmann, D., Heinze, S., Kurz, P., Bihlmayer, G. & Blügel, S. Resolving complex atomic-scale spin structures by spin-polarized scanning tunneling microscopy. *Phys. Rev. Lett.* **86**, 4132–4135 (2001).
